# An update on Peginterferon beta-1a Management in Multiple Sclerosis: results from an interdisciplinary Board of German and Austrian Neurologists and dermatologists

**DOI:** 10.1186/s12883-019-1354-y

**Published:** 2019-06-15

**Authors:** Annette Kolb-Mäurer, Cord Sunderkötter, Borries Kukowski, Sven G. Meuth, on behalf of the members of an expert meeting, Stefan Beissert, Gereon Nelles, Herbert Schreiber, Fritz Leutmezer, Til Menge, Christoph Lassek, Matthias Freidel, Tanja Stirnweiß

**Affiliations:** 1 Klinik und Poliklinik für Dermatologie, Venerologie und Allergologie, Universitätsklinikum Würzburg, Würzburg, Germany; 2 Universitätsklinik und Poliklinik für Dermatologie und Venerologie, Ernst-Grube-Str. 40, 06120 Halle (Saale) und Abteilung für translationale Dermatoinfektiologie, Röntgenstraße 21, 48149 Muenster, Germany; 3 Nervenärztliche Gemeinschaftspraxis, Groner-Tor-Straße 3, 37073 Göttingen, Germany; 4 Klinik für Neurologie, Universitätsklinikum Münster, Albert-Schweitzer-Campus 1, Gebäude A1, 48149 Münster, Germany

**Keywords:** Multiple sclerosis, Peginterferon bet-1a, Interferon beta, Flu-like symptoms, Injection site reactions, Management

## Abstract

**Background:**

Interferon (IFN) beta drugs have been approved for the treatment of relapsing forms of multiple sclerosis (RMS) for more than 20 years and are considered to offer a favourable benefit-risk profile. In July 2014, subcutaneous (SC) peginterferon beta-1a 125 μg dosed every 2 weeks, a pegylated form of interferon beta-1a, was approved by the EMA for the treatment of adult patients with RRMS and in August 2014 by the FDA for RMS. Peginterferon beta-1a shows a prolonged half-life and increased systemic drug exposure resulting in a reduced dosing frequency compared to other available interferon-based products in MS. In the Phase 3 ADVANCE trial peginterferon beta-1a demonstrated significant positive effects on clinical and MRI outcome measures versus placebo after one year. Furthermore, in the ATTAIN extension study, sustained efficacy with long-term treatment for nearly 6 years was shown.

**Main text:**

In July 2016, an interdisciplinary panel of German and Austrian experts convened to discuss the management of side effects associated with peginterferon beta-1a and other interferon beta-based treatments in MS in daily practice. The panel was composed of experts from university hospitals and private clinics comprised of neurologists, dermatologists, and an MS nurse. In this paper we report recommendations regarding best practices for adverse event management, focussing on peginterferon beta-1a. Injection site reactions (ISRs) and influenza-like illness are the most common adverse effects of interferon beta therapies and can present a burden for MS patients leading to non-adherence and discontinuation of therapy. Peginterferon beta-1a shows improved pharmacological properties. In clinical trials, the adverse event (AE) profile of peginterferon beta-1a was similar to other interferon beta formulations. The most common AEs were mild to moderate ISRs, influenza-like illness, pyrexia, and headache. Current information on the underlying cause of skin reactions associated with SC interferon treatment, and the management strategies for these AEs are limited. In pivotal trials, ISRs were mainly characterized and classified by neurologists, while dermatologists were only rarely consulted.

**Conclusions:**

This report addresses expert recommendations on the management of most relevant adverse effects related to peginterferon beta-1a and other interferon betas, based on literature and interdisciplinary experience.

## Background

Interferons are glycoproteins that have antiviral, antiproliferative, and immune regulatory functions. Since their initial description in 1957 [1], interferons have been increasingly used to treat a wide range of diseases worldwide. Interferon alpha was first approved in 1986 for the treatment of hairy cell leukemia and later in 1991 for hepatitis Non-A Non-B (now Hepatitis C). Non-pegylated interferon beta drugs were approved in the mid 1990s (EMA) for the treatment of relapsing MS (SC interferon beta-1b, 1995; intramuscular (IM) interferon beta-1a, 1997; SC interferon beta-1a, 1998). Since non-pegylated interferon betas are small proteins, they are rapidly cleared by the kidneys or degraded, and thus need to be given regularly, from once per week to every other day. Frequent parenteral application as well as AEs such as influenza-like illness and local injection site reactions (ISRs) might affect adherence to long-term therapy [2].

Pegylation is a well-established modification of therapeutic biologicals. It involves the covalent addition of a widely inert polyethylene glycol (PEG) moiety to therapeutic macromolecules, aiming at an improvement of their pharmacological properties. The PEG moiety increases the molecular mass of the drug, inhibits proteolysis, and reduces renal elimination, thus extending systemic drug exposure. Therefore, pegylated drugs usually enable significantly prolonged dosing intervals, with the potential added benefit of a more consistent exposure over time. Pegylation can be associated with reduced drug immunogenicity and side effects as antigenic epitopes are masked against recognition by immune cells and antibodies [3, 4].

Several pegylated drugs in different indications have been approved, including peginterferon alfa-2a and peginterferon alfa-2b. Pegylation of the interferon alfa molecules (alpha-2a, alpha-2b) improved the pharmacological properties of these proteins compared to the non-pegylated original forms, thereby boosting therapeutic efficacy (doubling sustained virologic response [SVR, no detectable viral HCV RNA in the blood 6 months after the completion of antiviral therapy] with peginterferon alfa versus interferon alfa) and reducing treatment frequency without increasing toxicity [5–7].

With the aim of yielding a modified, optimized interferon beta-1a molecule for the treatment of MS that requires less frequent dosing while maintaining the safety profile of the unmodified protein, pegylated interferon beta-1a (peginterferon beta-1a) was developed and later approved in July 2014 by the EMA (125 μg every 2 weeks) for the treatment of relapsing-remitting multiple sclerosis (RRMS). Phase 1 studies comparing the pharmacokinetics of peginterferon beta-1a with subcutaneous (SC) and intramuscular (IM) interferon beta-1a, respectively, demonstrated that peginterferon beta-1a provided a longer terminal half-life and greater systemic exposure compared with interferon beta-1a [8, 9].

ADVANCE was the pivotal 2-year, randomised, double-blind, parallel group, placebo-controlled (in year 1) Phase 3 trial of peginterferon beta 125 μg in patients with relapsing-remitting MS. Patients were randomized 1:1:1 to peginterferon beta-1a every 2 weeks, peginterferon beta-1a every 4 weeks, or placebo. At the end of year 1, all patients who received placebo were re-randomized to receive peginterferon beta-1a either every 2 or 4 weeks. In year 1, peginterferon beta-1a every 2 weeks (approved dose) demonstrated significantly higher clinical and MRI efficacy outcomes compared with placebo. Compared with year 1, annualized relapse rate (ARR) was lower in year 2. Patients starting peginterferon beta-1a every 2 weeks from year 1 displayed improved efficacy versus patients initially assigned to placebo, with reductions in ARR, 12-week disability progression, and 24-week disability progression (the latter was based on a post-hoc analysis and should be interpreted accordingly).

Over 2 years, greater reductions were observed with every 2 week versus every 4 week dosing for all endpoints [10]. In the ATTAIN extension study, relapse rates remained low up to nearly 6 years in the peginterferon beta-1a every 2-week group [11]. Studies with direct comparison of efficacy in MS patients treated with peginterferon beta-1a versus non-pegylated interferon beta preparations have not been conducted. Thus, definite conclusions on their relative efficacy cannot be drawn from the available evidence.

The safety profile of peginterferon beta-1a in the pivotal ADVANCE trial was consistent with those of non-pegylated interferon beta preparations for relapsing forms of MS. Peginterferon beta-1a was generally well tolerated, with the majority of adverse events being of mild or moderate severity. The most common AEs reported after one year were erythema (62% versus placebo 7%), influenza-like illness (47% versus placebo 13%), pyrexia (45% versus placebo 15%), and headache (41% versus placebo 33%) [12]. The safety profile of peginterferon beta-1a over 5 years was similar to year 1 in the ADVANCE trial [20].

The Phase 1 COMPARE study was conducted to not only provide a direct comparison of drug exposure, but also to assess comparative safety and tolerability of a single peginterferon beta-1a 125 μg dose versus 6 doses of SC interferon beta-1a (dosed at 44 μg, three times per week) over 2 weeks in healthy volunteers. The results demonstrated a significantly higher overall drug exposure (AUC_0-336h_ was 60%; *P* < 0.0001) with SC peginterferon beta-1a compared to SC interferon beta-1a. Peginterferon beta-1a dosing was associated with better tolerability, lower frequencies and incidence rates of ISRs, headache, myalgia and chills [9].

Although the results of the ADVANCE study show that ISRs and flu-like symptoms (FLS) associated with peginterferon beta-1a are mainly mild to moderate (95 and 90% respectively), and <  1% of patients discontinued treatment for this reason, these adverse events present a burden for MS patients and have a stronger impact on the patient’s daily life when compared to the randomized controlled trial setting. Hence, in July 2016, a panel of experts from university hospitals and private clinics comprised of neurologists, dermatologists, and an MS nurse convened in order to discuss the management of side effects associated with SC peginterferon beta-1a and other interferon beta-based treatments in MS. This article presents the outcome of the expert assessment of safety and tolerability of interferon beta-based therapies in MS as well as recommendations on their management in daily clinical care with a focus on ISRs (Table 1).

**Table 1:** Management of side effects

Strategies for the management of skin reactions	
• Patients should receive a detailed introduction on injection echniques and precautions to avoid cutaneous adverse events.	
• Warming the interferon solution to room temperature before injection.	
• Usage of an aseptic injection technique.	
• Rotation of the subcutaneous injection site at each injection.	
In the case of injection site erythema	
• Cold black-tea compresses can be applied for anti-inflammatory effects and cooling.	
• To reduce pruritus and erythema, topic treatment with 5% polidocanol may be applied.	
• Topical corticosteroids with appropriate therapeutic index in case of more severe erythema and eczema, palpable, infiltrated lesions, are e.g. prednicarbate (group II), mometasone (group III).	
• In order to achieve an additional itch-relief effect, lotions, creams and ointments can be stored in the refrigerator under hygienic conditions.	
Strategies for the management of FLS	
• Patients should be informed about the possibility of the occurrence and precautions to avoid or reduce FLS.	
• Gradual titration of the (Peg)interferon beta dose at the initiation of treatment according to the product information has been shown to significantly ameliorate symptoms.	
• Prophylactic and concurrent use of anti-inflammatory, analgesic and/or antipyretic treatments such as acetaminophen, ibuprofen, or naproxen may prevent or ameliorate FLSs.	
• Optimal timing of injection should be determined individually.	

## Main text

### Management of side effects associated with IFN beta therapy in MS

Recommendations by the authors and participating experts of the advisory board on mitigation strategies of adverse events (AEs) are discussed below in the context of published data and the respective Summary of Product Characteristics (SmPC). Population-based studies on the side-effect profile in sizeable populations treated in the routine treatment setting have not been published to date. Therefore, the content of this report is based on the available literature as of December 2017^1^ and personal opinion or communication.

#### Skin reactions

**Fig. 1:**
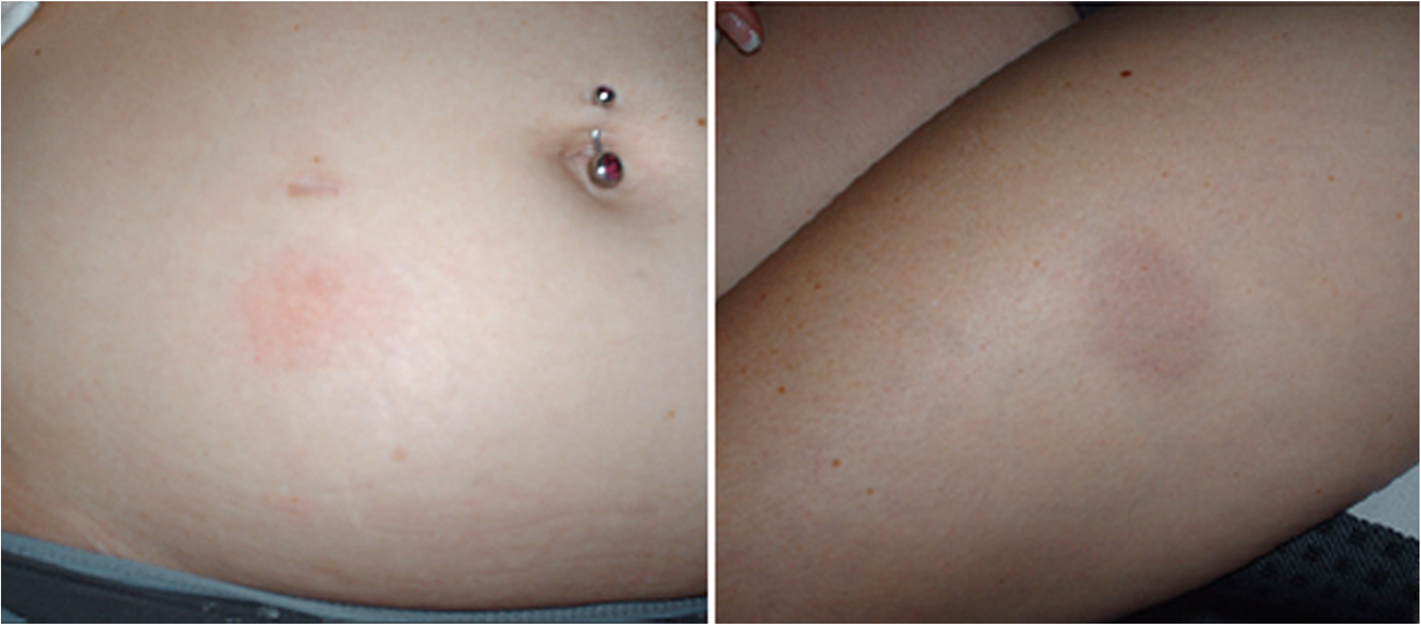
Erythema observed in an adult patient injecting peginterferon beta-1a. (Image published with permission from expert. Patient consent was obtained for publication)

Injectable disease-modifying therapies (DMTs) can be associated with local skin reactions at the injection site and systemic cutaneous side effects. The pattern and incidence of ISRs appear to be dependent on the type of interferon (e.g. peginterferon beta-1a, interferon beta 1a, interferon beta 1b) and route of administration (SC versus IM injection). In general, injection site reactions may present as erythema, bruises, swelling, induration, pain, pruritus, eczema-like reactions, infection, abscess, lipoatrophy or necrosis (Table 2, Fig. 1).

**Table 2:** Skin reactions reported under therapy with peginterferon beta-1a, interferon beta 1a and 1b, respectively

Frequency according to SmPC of respective drug	SC peginterferon beta-1a [19]	IM interferon beta-1a [21]	SC interferon beta-1a [22]	SC interferon eta 1b [23]
Very common ≥1/10	IS erythema, IS pain, IS pruritus		IS inflammation, IS skin disorder reaction	IS reaction (different kinds), sweating
Common ≥1/100, < 1/10	IS oedema, IS warmth, IS haematoma, IS rash, IS swelling, IS discolouration, IS inflammation	Rash, increased sweating, contusion, IS pain, IS erythema, IS bruising	Pruritus, rash, erythematous rash, maculo-papular rash, alopecia, IS pain	Erythema, swelling, inflammation, pain, abscess, skin disorder, rash, urticaria, pruritus, alopecia, IS necrosis
Uncommon ≥1/1.000, < 1/100	Urticaria, hypersensitivity reaction	Alopecia, IS burning	Urticaria, IS necrosis, IS swelling, IS infections*, increased sweating	Skin discolouration
Rare ≥1/10.000, < 1/1.000	IS necrosis		Quincke’s oedema (angio-oedema), erythema multiforme, erythema, multiforme-like skin reactions, Stevens Johnson syndrome, IS cellulitis, anaphylactic reactions	
Unknown		Angioneurotic oedema, pruritus, rash vesicular, urticaria, IS reaction, IS inflammation, IS cellulitis, IS necrosis, IS bleeding, anaphylactic reactions, hypersensitivity reaction (angio-oedema, dyspnea, urticarial, rash, pruritus)		Drug induced lupus erythematodes

Low grade cutaneous reactions are observed in up to 90% of patients treated with subcutaneously administered interferon beta preparations (compared to 20% of patients using IM interferon beta-1a) [13, 14]. More severe skin reactions including cutaneous infections, deep ulcerations or necrosis are observed at low frequencies (1–5%) [14, 15]. Generally, IM injections cause less skin reactions than SC injections, but cases with necrosis have also been reported for interferon beta-1a IM [14, 21].

In studies aiming at the identification of risk factors predisposing for skin reactions to injected interferon beta preperations, no correlations with preexisting atopic dermatitis, body mass index, gender or application via an autoinjector device ere observed. Lipoatrophy was observed somewhat more frequently in females versus male patients [15, 16]. In general, local ISRs are seen more frequently in skin areas with less subcutaneous fat [17].

In contrast to local cutaneous side effects as described above, systemic immune-mediated and inflammatory cutaneous adverse events are rare [15]. Interferons have not been shown to play a definite causal role in most reported cases, thus it is unclear whether they truly contribute to the development of immune-mediated cutaneous adverse events [15].

In the ADVANCE trial, erythema, pain, and pruritus were the most commonly observed ISRs associated with peginterferon beta-1a therapy [12]. Of the patients who experienced ISRs, 95% reported them as mild or moderate in severity. The described onset of erythema under peginterferon beta-1a was often delayed following injection, typically by more than 24 h (ca. 1–4 days) in about 90% of patients [18]. The reported duration of ISR, usually more than 3 days, was longer than most patient experienced with non-pegylated interferon beta drugs [18]. However, erythema associated with peginterferon beta-1a treatment has not been demonstrated to be disruptive to patients’ daily activities [18]. One patient experienced an injection site necrosis with SC peginterferon beta-1a in the pivotal ADVANCE trial, which was resolved with standard therapy. Hypersensitivity reactions occurred in 16% of patients receiving peginterferon beta-1a (14% of patients with placebo), and <  1% of peginterferon beta-1a recipients experienced a serious hypersensitivity event (e.g. angioedema, urticaria). These patients recovered promptly following anti-histamine and/or corticosteroid therapy [19].

In the population of the extension study ATTAIN, injection-site reactions were less frequent in year 3–6 of therapy (43%) vs. year 1–2 (70%). Specifically, erythema, pain and pruritus were clearly reduced in the extension period vs. the double-blind phase [20]. Thus, it appears that the prevalence of local reactions declines with prolonged use of peginterferon beta-1a.

#### Mitigation of injection-site reactions

The expert panel recommended that before starting therapy, patients should be informed about the safety and tolerability profile of the injectable MS therapy. Patient education about the possibility that SC drugs may cause ISRs is an especially effective strategy to diminish anxiety when it occurs, as confirmed by two DELPHI surveys [18, 24], particularly when the onset of ISRs is delayed after the application. Illustrations showing the potential range of occurring erythema can help to set expectations. As recommended within the SmPC of the respective drug, healthcare professionals should train patients in the proper technique for self-administering subcutaneous injections to minimize cutaneous adverse events. As a practical measure, the experts suggested that patients should avoid injecting cold solution (directly out of the refrigerator) to reduce injection-related discomfort, and consequently the drug should be warmed to room temperature prior to injection [25]. The injection should not be applied in areas of the body where the skin is irritated, reddened, bruised, infected, tattooed or scarred in any way. The expert panel agreed with the label recommendations specifying that patients should also be advised to regularly rotate and not use the same injection site for consecutive injections [19].

The expert panel was of the opinion that pen devices and other autoinjectors have improved the convenience of administration and facilitate self-injection. They have reduced the burden of injectable MS-therapies and diminished problems associated with needle phobia and inadequate injection techniques. However, some patients still prefer to use the prefilled syringe instead of an injection device. A few of these patients report that they observed significantly less local cutaneous side effects when compared to autoinjector usage, independent of the interferon beta preparation [personal observation]. Patients who are apprehensive of SC injection might inject intradermally instead of subcutaneously due to a timid and halting injection procedure with an automatic administration device. In case of accidental intradermal injection (e.g. due to patient anxiety), the adverse skin reaction at the injection site may be increased. The prefilled syringe provides an alternative for patients who want to control the self-injection and are at risk of discontinuing therapy due to the autoinjector.

Patient support programs (PSP) with specialized nurses or skilled healthcare professionals have shown to enable continuous patient education and consistently confirmed treatment benefits [26]. The experts recommend to take advantage of special patient support programs or patient care by MS nurses. Accompanying patients in everyday practice may have strong positive psychological effects in some patients. Furthermore, MS patients are being informed on healthy skin practices and should be able to identify for signs and symptoms of ISRs or at least have the possibility to approach MS-nurses or specialized contact health care professionals within the PSP.

### Erythema, pruritus, swelling, and pain

Injection-site pain, erythema, swelling, and pruritus may be reduced by applying warm compresses before injection and cold compresses after injection for up to 5 min. The expert panel emphasized that the uses of ice packs for cooling of the affected areas is generally not advised, as it can lead to local frostbites. The tissue can be relaxed by warming or slightly massaging the area to improve medication absorption, prevent inflammation, and improve circulation so that the medication can be more easily dispersed from the area. A pilot study has been initiated to examine whether warm and cold compresses reduce injection site erythema due to peginterferon beta-1a [27]. Theoretically, both warm and cold modalities may improve interferon injection tolerance. Application of warm compresses before injection may increase local blood flow and rapid systemic absorption, while applying cold compresses after injection may reduce erythema, edema, and pain that often follows SC injections.

As recommended within the SmPC of the respective drug and reported previously, the site of administration should be disinfected (e.g. with an alcohol wipe) and dried before dosing [25].

Properly applied black-tea compresses can have an additional anti-inflammatory effect, and was endorsed by the dermatologists in the expert panel. Brewed unflavored black tea should steep for at least 20 min, be chilled to the desired temperature (e.g. in the refrigerator under hygienic conditions), and placed on the affected skin with a single layer of linen cloth.

For local itch relief and redness, dermatologists of the expert panel recommend the drug polidocanol, which is a local anesthetic and antipruritic alkyl-polyglycolether that is used in the treatment of itching skin conditions, e.g. eczema [27, 28]. In the case of skin reactions that occur after administering SC interferons, the expert panel dermatologists recommend the use of 5% polidocanol as a formulation or as a component of a ready-to-use preparation. The application can be conducted as needed up to 5–10 x per day.

Local antihistamine administration (e.g. dimetinden maleate), on the other hand, can cause dehydration of the skin and trigger allergies [29]; therefore, it was not recommended for mitigation of skin reactions at the injection site by the expert panel. When itching is very prominent, oral antihistamines were recommended instead by the expert panel; during the day, non-sedating antihistamines (e.g. desloratadine) and in the evening, optionally, sedating antihistamines (e.g. clemastine) were preferred.

Arnica preparation may cause contact dermatitis (allergies) which is usually attributed to the herbal compounds sesquiterpene lactones that may also have some anti-inflammatory properties [32]. Arnica is therefore less suitable and was not recommended by the dermatologists in the expert panel for management of local skin reactions after injections.

The application of vitamin K cream at the injection sites of non-pegylated interferon beta drugs has been show to reduce local reactions [31].

### Severe skin reactions, rash, and eczema

In the case of severe skin reactions, rash and eczema-like reactions, higher-potency topical glucocorticoids of classes 2 to 4 (e.g. prednicarbate, mometasone or clobetasol) that have a good therapeutic index may be applied for 2–3 days post-injection (even on a large area, if necessary), as recommended by the dermatologists in the expert panel. In addition, as an occlusive treatment, the affected area may be enclosed overnight after treatment with the above glucocorticoids, e.g. with a plastic wrap or a waterproof bandage. As a result, the active substance penetrates into deeper skin layers. This technique may also be helpful in patients with recurrent skin reactions.

Topical hydrocortisone preparations are usually insufficient to manage inflammation and to mitigate injection site erythema.

### Skin infections or necrosis

Severe skin reactions such as deep ulceration, infection or necrosis are rare adverse events and more likely seen under interferon beta-1b treatment as reported in the literature [14].

The pathogenesis of skin ulcerations and necrosis is still unclear. As discussed by the expert panel, unintended injection into deeper skin layers, e.g. into or around an artery can potentially lead to severe vasospasm (*embolia cutis medicamentosa*). It is also possible that the vessels that supply the arterial wall become punctured, triggering thrombotic occlusions. Patients usually report severe pain directly after the injection, followed by necrosis over the course of the following hours. Management of necrosis may require surgical treatment or antibiotics if infected.

One possible underlying cause for necrosis could be an immune complex vasculitis since an association between the occurrence of neutralizing antibodies to SC interferon beta and necrotizing skin lesion have been reported, [30], however, a genuine immune complex vasculitis would require palpable and retiform purpura in dependent body parts.

### Post-inflammatory hyperpigmentation

A formation of hyperpigmentation is occasionally observed at sites of peginterferon beta-1a injection. Patients with this type of skin reaction, i.e. post-inflammatory hyperpigmentation, should avoid exposure of the affected skin to direct sunlight and UV irradiation. The hyperpigmentation may take several months or years to fade or may persist at sites where more severe local inflammatory reaction to the drug had occurred [personal observation].

#### Flu-like symptoms

FLS that include a variable combination of fever, myalgia, headache, asthenia, fatigue, and chills account for the most common adverse event in interferon-beta treated patients. The individual pattern and intensity of these symptoms also varies considerably. Incidence rates associated with non-pegylated interferon beta drugs have ranged from 40 to 61% in pivotal clinical trials [10, 12, 33–35]. The safety profile of peginterferon beta-1a observed in clinical trials and after approval was consistent with profiles observed after non-pegylated interferon beta treatments for relapsing MS [personal observation]. Next to ISRs, FLS are the most common side effects and include influenza like illness, pyrexia, headache, myalgia, chills, asthenia, and arthralgia. In the ADVANCE trial, influenza-like illness was experienced by 47% of patients receiving peginterferon beta-1a, and 13% of patients receiving placebo after 1 year [10, 12]. Of the patients who reported FLS with peginterferon beta-1a dosed at 125 μg every 2 weeks, 90% reported them as mild or moderate in severity. None were considered serious in nature. Less than 1% of patients who received peginterferon beta-1a during the ADVANCE study discontinued treatment due to FLS after 1 year [19].

Patients are more likely to experience FLS when initiating interferon beta therapy. The severity and incidence tend to decrease over time after continued therapy: In the population of the extension study ATTAIN, FLS were less frequent in year 3–6 (43%) vs. year 1–2 (53%). Fever in particular was observed in only 24% of the patients during the extension period vs. 50% in the double-blind phase [20].

FLS with non-pegylated IFN beta treatments typically occur relatively soon after the application, e.g. about two to six hours after injection, and usually improve within 24 h [17].

The median time to FLS onset after injection of peginterferon beta-1a in patients switching from interferon beta therapy to peginterferon beta-1a was 10 h (interquartile range, 7 to 16 h) and the median duration was 17 h (interquartile range, 12 to 22 h) [19]. With peginterferon beta-1a injection every 2 weeks, the occurrence of FLS over the year is reduced due to the reduced frequency of injections versus non-pegylated interferon beta therapies.

As reported previously, the initiation phase of interferon treatment is the most critical period and can affect patients’ views on the long-term acceptability of their therapy [17]. This shows the importance for mitigation strategies to reduce treatment-related adverse events such as FLS during the interferon initiation phase. In order to manage potential FLS, the experts also emphasized the benefit of special patient support programs or patient care by MS nurses. Patient education by MS nurses or a specialized health care professional within the PSP are also cited by the expert panel as essential for managing FLS.

### Titration and prophylactic co-medication

The development of FLS is related to an interferon beta-induced secretion of cytokines and mediators such as interleukin 6 (IL-6), interferon gamma, and prostaglandin levels. Detailed strategies to manage FLS in MS patients have been established including several nonpharmacological and pharmacological approaches [24].

There is no evidence-based guideline for the best time of the day to administer interferon beta because the pivotal efficacy studies conducted in the 1990s did not collect data regarding this issue. A consensus statement published in 1996 [36], however, recommends administering interferon beta in the evening before bedtime. The patient may sleep through the symptoms instead of experiencing them during active daytime periods. Alternatively, injecting in the morning can qualitatively improve interferon beta-related FLS and sleep [37]. However, since median FLS onset after peginterferon beta-1a treatment is 10 h after injection and is widely variable between patients (interquartile range between 7 and 16 h), the experts agreed that the timing of the peginterferon beta-1a injection every 2 weeks should be considered individually [19].

Furthermore, the expert panel was in accordance with the label recommendations specifying that prophylactic and concurrent use of anti-inflammatory, analgesic and/or antipyretic treatments such as acetaminophen, ibuprofen, or naproxen may prevent or ameliorate FLS sometimes experienced during interferon treatment [19].

The expert panel agreed that gradual titration of the interferon beta dose at the initiation of treatment according to the product information [19] has been shown to significantly ameliorate symptoms.

#### Psychiatric disorders – depression

The prevalence of psychiatric disorders is increased in MS [38]. The lifetime prevalence of major depressive disorder is higher compared to the general population (36–54% versus 16.2%), with an estimated doubling of suicide rates [39].

The association of depressed mood and disease-modifying therapies, particularly interferon beta remains unclear [41, 43]. While no significant increase in depression was found by several authors for IFN beta versus control groups [38–40] other authors observed higher rates in pateints receiving interferons [42–46].

In the ADVANCE study, the incidence of depression during the first year of study was similar in patients receiving peginterferon beta 1a compared to the placebo group. The overall incidence of depression or suicidal ideation was 8% for both peginterferon beta-1a and placebo [19]. Serious events of depression and suicidal ideation occurred infrequently (< 1%) in both groups.

An association of depression with interferon beta drugs remains a matter of debate, while depressive symptoms are generally observed more frequently in MS patients. The German DGN therapeutic guidelines recommend that affected patients should be observed more closely and early antidepressive therapy should be considered. The SmPCs of all interferon beta drugs include a warning regarding an increased frequency of depression in MS patients in general and in association with the use of interferon beta preparations. Thus, all interferon beta drugs and peginterferon beta-1a should be used with caution in MS patients with a history of depressive disorder. All interferon beta-based drugs are contraindicated in patients presenting with severe depression and/or suicidal ideation [19–23]. The expert panel supports the recommendations of the gudelines.

#### Laboratory abnormalities

The use of interferons can be associated with laboratory abnormalities. The most commonly observed laboratory abnormalities that occur after the initiation of an interferon beta-therapy are lymphopenia, neutropenia, leukopenia, and raised liver aminotransferase values [17, 47].

#### Hematologic adverse effects

Hematologic adverse effects observed in patients receiving interferon beta preparation are generally mild, transient and self-limiting. The underlying mechanism is thought to be related to bone marrow [48, 49].

The healthy participants of a phase I study (*n* = 69), received a single dose of peginterferon beta-1a (63 μg, 125 μg, 188 μg; injected SC or IM) or IFN beta-1a (30 μg IM), respectively [8]. During follow-up, the cell numbers of lymphocyte subpopulations (T cells, CD4+ and CD8+ cells, B cells, NK cells) decreased transiently on both tested drugs. Nadir levels were reached at 12 h post-injection with non-pegylated interferon beta-1a (reduction by 15 to 92%) and between 12 and 24 h post-injection with peginterferon beta-1a (reduction by 9 to 92%). The cell counts returned to baseline levels within 36 h after injection in the interferon beta-1a group. In the peginterferon beta-1a groups, baseline levels were reached at 7 days after injection, except for B cells that returned more slowly, with partial recovery observed by 28 days after application.

#### Hepatic adverse effects

Abnormal transaminase [alanine transaminase (ALT), aspartate transaminase (AST)] and bilirubin levels have been reported in patients receiving interferon beta-therapy [49; FIs Betaferon; Plegridy]. Elevated serum hepatic enzyme levels, hepatitis, autoimmune hepatitis and hepatic failure has been reported in the post-marketing phase with interferon beta treatments. In some cases, these reactions have occurred in the presence of other co-medications (e.g. ibuprofen, naproxen, indomethacin) that have been associated with hepatic injury.

Hepatic monitoring is recommended and should be performed together with the other required laboratory monitoring (prior to initiation, in regular intervals following start of therapy, periodically thereafter in the absence of clinical symptoms).

#### Effects on thyroid function

Autoimmunity and conditions associated with thyroid dysfunction such as hypothyroidism and hyperthyroidism have been observed with the use of interferon beta products. A review of the literature revealed an overall mean prevalence of incident thyroid dysfunction of 6.2% with hypothyroidism occurring more frequently (3.9%) than hyperthyroidism (2.3%) [50]. Thyroid disease has been reported especially in treated patients with pre-existing autoimmunity. Although autoimmune reactions or immune system dysregulation provide the most likely explanation for the occurrence of thyroid disease with type I interferon therapy, a direct inhibitory effect on thyrocytes may be presumed in patients who developed hypothyroidism without autoimmunity [50].

Regular thyroid function tests are recommended in patients with a history of thyroid dysfunction or as clinically indicated.

The expert panel recommended to follow monitoring rules according to the product information and local guidelines [51, 52]: In addition to laboratory tests normally required for monitoring patients with MS, complete blood and differential blood cell counts, platelet counts, and blood chemistries including liver enzyme tests (e.g. AST, ALT), renal and thyroid function are recommended prior to initiation, at regular quarterly intervals within the first year following introduction of interferon therapy and then periodically thereafter in the absence of clinical symptoms, 1-2x per year (Table 3) [51, 52]. Patients with myelosuppression may require more intensive monitoring of complete blood cell counts, including differential and platelet counts.

**Table 3:** Laboratory and MRI monitoring of interferon beta patients according to the German competence network multiple sclerosis (KKNMS) [52]

	Prior to IFN initiation	During IFN treatment		
		After 1 month of initiation	Quarterly during first year of treatment	1 year of initiation: every 6–12 months or as clinically indicated
Complete, differential blood cell & platelet counts	x	x	x	x
Liver enzyme tests (ALT, AST, GGT, bilirubin)	x	x	x	x
Renal function (creatinine, estimated creatinine clearance/GFR)	x	x	x	x
Inflammation and Infections (urine status)	x			
TSH		periodically		
Pregnancy test	x			
MRI	x	annually		

## Conclusions

In July 2014, peginterferon beta-1a, the first pegylated interferon beta-1a molecule, was approved by the EMA for the treatment of adult RRMS patients. Interferon beta therapies have been used for more than 20 years in the treatment of MS and have established themselves as effective and safe therapeutic options.

Peginterferon beta-1a allows for prolonged application intervals of 2 weeks with a safety and tolerability profile that appears largely similar to those of the non-pegylated interferon beta preparations. The prolonged dosing interval may support long-term adherence and persistence to the prescribed regimen.

The most common adverse events are mild to moderate erythema at the injection site and presence of FLS, although the latter occurs less frequently. The onset of FLS usually starts at 10 h after the injection, whereas FLS that occur after injection of non-pegylated interferon beta usually begin after about 6–8 h. The management of interferon beta side effects has been established; for FLS, titration at the start of therapy, prophylactic and concurrent anti-inflammatory, analgesic and/or antipyretic co-medication as well as individual injection time is recommended. For ISRs, proper self-administration technique, rotation of the injection sites, warming of the injection solution to room temperature, cooling of the administration site after injection, and the use of topical preparations for potential ISRs are recommended.

The expert panel emphasized that educating patients (both treatment naïve and switchers) on the characteristics and management of potential adverse events, e.g. FLS and ISRs, before starting treatment is considered important for setting treatment expectations, promoting persistence/adherence, and improving treatment outcomes. During interferon treatment, the expert panel agreed that patients should be monitored as recommended by the SmPC and local guidelines.

Intensive patient care by the MS nurse or in special patient support programs has a strong psychological effect while accompanying patients in everyday practice.

Overall, there is more than 20 years of long-term safety and efficacy experience with interferons in patients with RRMS. Peginterferon beta-1a, with reduced frequency of injections, hence constitutes an attractive new option for the treatment of RRMS with an established benefit-risk ratio.

## Data Availability

Not applicable.

## Abbreviations

AEs: Adverse events
ALT: Alanine aminotransaminase
AST: Aspartate aminotransferase
DGN: German Society of Neurology (Deutsche Gesellschaft für Neurologie)
DMTs: Disease-modifying therapies
EMA: European Medicines Agency
FDA: US Food and Drug Administration
FLSs: Flu-like symptoms
GFR: Glomerular filtration rate
GGT: Gamma-glutamyl transferase
HCV: Hepatitis C virus
IFN: Interferon
IM: intramuscular
KKNMS: German competence network multiple sclerosis (Krankheitsbezogenes Kompetenznetz Multiple Sklerose)
IS: Injection site
ISRs: Injection site reactions
MRI: Magnetic resonance imaging
MS: Multiple sclerosis
PSP: Patient support program
NK: Natural killer
RMS: Relapsing multiple sclerosis
RRMS: Relapsing remitting multiple sclerosis
SC: Subcutaneous
SmPC: Summary of product characteristics
SVR: Sustained virologic response, no detactable viral HCV RNA 6 months after end of therapy
μg: Microgram

## Footnotes

1 ^1^Literature searches were performed in the PubMed database using “interferon(s)”, “multiple sclerosis”, “MS”, “flu-like” and “FLS” as search terms.
